# The Platelet Anaphylatoxin Receptor C5aR1 (CD88) Is a Promising Target for Modulating Vessel Growth in Response to Ischemia ^a^

**DOI:** 10.1055/a-2156-8048

**Published:** 2023-10-19

**Authors:** Henry Nording, Lasse Baron, Antje Lübken, Hossein Emami, Jacob von Esebeck, Moritz Meusel, Christian Sadik, Nancy Schanze, Daniel Duerschmied, Jörg Köhl, Götz Münch, Harald F. Langer

**Affiliations:** 1 Cardioimmunology Group, Medical Clinic II, University Heart Center Lübeck, Lübeck, Germany; 2 DZHK (German Centre for Cardiovascular Research), Partner Site Hamburg/Lübeck/Kiel, Lübeck, Germany; 3 Medical Clinic II, University Hospital, University Heart Center Lübeck, Lübeck, Germany; 4 Clinic for Dermatology, University of Lübeck, University Hospital, Lübeck, Germany; 5 Department of Cardiology, Angiology, Haemostaseology and Medical Intensive Care, University Medical Centre Mannheim, Medical Faculty Mannheim, Heidelberg University, Mannheim, Germany; 6 German Centre for Cardiovascular Research (DZHK), Partner Site Heidelberg/Mannheim, Germany; 7 European Center for Angioscience, Medical Faculty Mannheim, Heidelberg University, Mannheim, Germany; 8 ISEF, University of Lübeck, Lübeck, Germany; 9 AdvanceCOR, Martinsried, Germany

Diseases featuring tissue ischemia are wide-spread and among the main cause of death in the Western world. 1 To date, the exact mechanisms of revascularization of tissues after arterial obstruction, usually as a result of atherosclerosis, 2 remain incompletely understood. This is underscored by the fact that pharmacological approaches to enhance vessel growth in patients with chronic vascular occlusions are not successfully established for clinical use, yet. 3 Several reports implicate complement activation as a contributor to ischemic tissue injury via interaction with reactive oxygen species, 4 for example, the lectin 5 or the classical pathway. 6 7 Thus, the complement system may be a possible therapeutic target in this disease setting (reviewed in Markiewski et al 8 ).

The complement system is a very well-preserved and phylogenetically old part of the immune system serving several functions from immune protection to tissue homeostasis and dysfunction. 9 Previously, the complement receptor C5aR1 was demonstrated to be pro-angiogenic, 10 but has also been attributed an inhibitory role for neovascularization. 11 Interestingly, platelet activation can colocalize with areas of increased complement activity and several functional links have been described connecting complement and platelets. 12 13 14

Platelets play a decisive role in cardiovascular diseases featuring thrombosis, where they are the decisive cellular part of any thrombus and contribute to several mechanisms of thrombus formation. 15 Beyond that, platelets were recently implicated in tissue remodeling processes such as apoptosis, 16 17 immune patrolling, 18 or adaptive immunity. 19 Furthermore, platelets contribute to the immediate response after vascular injury by promoting vascular inﬂammation, 15 immunomodulation, 20 21 22 and atherosclerosis. 23 24 Recently, we could show that complement receptors are expressed on platelets, and that the anaphylatoxin C3a receptor modulates primary hemostasis. 25 26 Moreover, platelets express C5a receptor 1 (C5aR1). 27 Absence of this anaphylatoxin receptor, however, had no effect on in vivo thrombus formation. 25 Thus, it might be of alternative fictional relevance for platelet effects in tissue remodeling.

The group of J. Italiano could visualize that platelets store pro- and antiangiogenic factors in distinct granules and can release them upon stimulation. 28 Recently, we demonstrated that C5aR1-induced CXCL4 release modulates revascularization. 14 Here, we present further data on the importance of C5aR1 on platelets for the modulation of tissue revascularization.

**Fig. 1:**
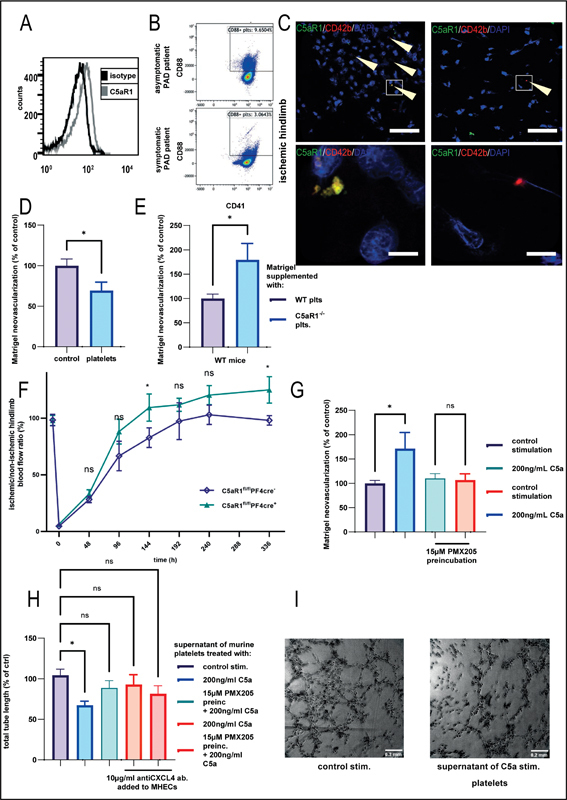
( **A** ) Washed murine platelets were stained with a C5aR1-specific antibody or isotype control. Displayed is a histogram representative of four independent platelet samples. ( **B** ) In patients with peripheral artery disease (PAD), C5aR1 expression was increased in asymptomatic disease versus symptomatic PAD patients at Fontaine stage IIb. Shown are representative images of 20 patients. ( **C** ) Immunofluorescence co-staining of ischemic murine hindlimb gastrocnemius muscle sections at 630× magnification showing colocalization of the platelet markers CD42b (red) and C5aR1 (green) 1 week after induction of ischemia. Representative images confirm that the cells are anuclear (DAPI-negative, blue). Arrows point to platelets. Scale bars represent 5 µm. ( **D** ) In vivo Matrigel plug assay experiments were performed using WT mice. Matrigel was supplemented with bFGF and freshly isolated platelets from WT mice or vehicle control. After 7 days, Matrigels were explanted, and vessel density way assessed by H&E staining. We observed decreased vessel growth after addition of isolated platelets. Data are displayed as % of control. *n* = 6–7 plugs were analyzed. The group with vehicle control represents 100%. * *p* < 0.05. ( **E** ) Similarly, Matrigel was supplemented with bFGF and freshly isolated platelets from WT or C5aR1 ^−/−^ mice were additionally injected. After 7 days, Matrigels were explanted, and vessel density was assessed by H&E staining. We observed increased vessel growth in the absence of the C5aR1 on platelets. Data are displayed as % of control. *n* = 7 plugs were analyzed. Data are displayed as % of control. The group with WT mice represents 100%. * *p* < 0.05. ( **F** ) Mice bearing a platelet-specific knockout of C5aR1 on platelets were generated (PF4-cre ^+^ C5aR1 ^fl/fl^ ). PF4-cre ^+^ C5aR1 ^fl/fl^ and PF4-cre ^−^ C5aR1 ^fl/fl^ mice were subjected to hindlimb ischemia and analyzed over 2 weeks. Revascularization of the hindlimbs after femoral artery ligation was visualized by laser Doppler fluximetry (LDI). We found increased revascularization in PF4-cre ^+^ C5aR1 ^fl/fl^ compared with PF4-cre ^−^ C5aR1 ^fl/fl^ animals, although this reached statistical significance only at some time points. Data are shown as the mean ± SEM ( *n* = 3 animals per group) and are displayed as % of the perfusion in the contralateral control limb. * *p* < 0.05. ( **G** ) As prior experiments suggested a platelet-mediated mechanism of C5aR1 control of revascularization, we stimulated washed human platelets with C5a. The supernatant was analyzed by ELISA for the level of CXCL4. Preincubation with the C5aR1 inhibitor PMX205 inhibited C5a-induced CXCL4 secretion. Data are shown as the mean ± SEM. *n* = 4–8. The group with WT mice represents 100%. * *p* < 0.05. ( **H** ) Platelets from WT mice were stimulated with C5a or control and preincubated with PMX205 or control. The supernatant was co-incubated with endothelial cells (MHEC-5T). In some groups, an anti-CXCL4 antibody was added to the endothelial cells. C5a-stimulated WT platelet supernatant inhibited endothelial tube formation, which was not detectable when C5aR1 or CXCL4 was inhibited. Data represent mean ± SEM of the total tube length. Data are displayed as % of control and the group with control stimulated supernatant treatment represents 100%. *n* = 10–11. * *p* < 0.05. ( **I** ) Representative images of endothelial tube formation show the inhibitory effect of C5a-conditioned platelet supernatant compared with control. SEM, standard error of the mean; WT, wild type.

First, we analyzed C5aR1 expression on murine platelets ( Fig. 1A ). Then, we assessed receptor expression levels on platelets in a cohort of peripheral artery disease patients, which were not symptomatic. Previously, this condition has been linked to improved formation of collateral vessels, and presumably thereby lack of pain symptoms. 29 Interestingly, we observed an increased expression of C5aR1 on platelets of patients with an asymptomatic disease ( Fig. 1B ). This led us to investigate the platelet C5aR1 in the hindlimb ischemia model. In this mouse model of ischemic disease, the femoral artery is ligated to induce tissue ischemia (see Supplementary Materials for further details). One week after induction of tissue ischemia, the distal gastrocnemius muscle was explanted and processed for immunofluorescence microscopy. Co-staining of CD42b (red, “a platelet marker”) and C5aR1 (green) revealed expression of C5aR1 on DAPI (blue)-negative platelets in the ischemic tissue ( Fig. 1C ). To decipher the role of platelets in a controlled proangiogenic environment, we used a further mouse model, the Matrigel plug assay, whereby a gel-like substance is injected into the skin of mice forming an extracellular-space-like matrix. If the matrix is supplemented with vascular growth factors such as bFGF, vessels grow into the plugs, which can then be quantified. We injected Matrigel supplemented with bFGF and freshly isolated platelets with (wild type [WT]) or without C5aR1 (C5aR1 ^−/−^ ) into C57/Bl6 mice. Addition of platelets to the Matrigel resulted in reduced bFGF-mediated angiogenesis ( Fig. 1D ). Interestingly, presence of C5aR1 on WT platelets was associated with a reduced extent of vessel growth into the plugs and supplementation with C5aR1 ^−/−^ platelets resulted in significantly more revascularization ( Fig. 1E ).

We then created a platelet-specific C5aR1-deficient mouse strain using the cre-lox system (for details please refer to the Material and Methods section in the Supplementary Information 14 ). We assessed platelet reactivity by stimulating diluted whole blood with collagen-related protein and measured the platelet marker β _3_ integrin (GPIIIa, CD61), which mediates binding to fibrinogen. There was no significant difference in between both strains ( Supplementary Fig. S1A ), suggesting that there are no effects on the fibrinogen receptor. However, addition of C5a induced some activation in platelets on the level of fibrinogen binding and activated GPIIbIIIa (measured by activation-specific PAC1 antibody), but not on P-selectin upregulation ( Supplementary Fig. S2 ). These effects could be blocked by the C5aR1 antagonist PMX205 ( Supplementary Fig. S3 ). In the absence of C5a ligation, WT and C5aR1 ^−/−^ platelets did not display differences in binding to fibrinogen ( Supplementary Fig. S4 ). We monitored revascularization over 14 days post induction of hindlimb ischemia and observed that the C5aR1 ^flox/flox^ PF4cre ^−^ mice show a lower degree of revascularization than the C5aR1 ^flox/flox^ PF4cre ^+^ littermates, where C5aR1 is absent in platelets ( Fig. 1F ). Thus, the platelet C5aR1 is an important modulator of revascularization.

As all previous data suggest a platelet-mediated effect of C5aR1 on platelets, we stimulated platelets from WT mice with C5a in vitro. Using an ELISA-based analysis, we could measure a release of antiangiogenic CXCL4 (platelet factor 4, PF4) from platelets upon C5a stimulation, which could not be observed when C5aR1 was blocked by the C5aR1 antagonist PMX205 ( Fig. 1G ). To verify that the observed effect is indeed mediated through CXCL4 secreted from platelets, we assessed endothelial tube formation in vitro and co-incubated cells with C5a-conditioned platelet supernatant ( Fig. 1H, I ). Confirming our hypothesis, we could show that C5a-conditioned platelet supernatant inhibited endothelial tube formation, which could be blocked by administering the C5aR1 antagonist PMX205 ( Fig. 1H ). Interestingly, a neutralizing antibody against CXCL4 did not have an additive effect on top of PMX205 in reversing the effect of C5a-conditioned platelet supernatant ( Fig. 1H ). As the collagen pathway of platelet activation has gained recent attention, 30 we excluded involvement of glycoprotein VI in C5a-mediated release of CXCL4 from platelets ( Supplementary Fig. S5 ). Together, we demonstrate that C5a-mediated release of CXCL4 is one mechanism how platelets modulate vessel growth.

Here, we identified a novel mechanism for inhibition of neovascularization via CXCL4 secretion induced by platelet C5aR1. Targeting the complement system may offer novel approaches to treat patients with diseases featuring tissue ischemia and inflammation. These diseases are largely caused by atherosclerosis. 2 Interestingly, the anaphylatoxin receptors C5aR1 and C3aR are expressed in atherosclerotic plaques. 31 Importantly, in patients with atherosclerosis, expression of C3aR and C5aR shows a signiﬁcant correlation with platelet activation markers. 25 27 32 In a mouse model, anti-C5aR1 compounds have already proven effective in enhancing revascularization. 14 The potential for the treatment of human patients, however, will first have to be evaluated in future clinical studies.

Recently, the C5a–C5aR1 axis was suggested to be an important marker of inflammation associated with COVID-19. 33 Interestingly, the platelet C5aR1 has been recognized as an important player in the complex pathophysiology of SARS-CoV-2 infection. 34 Thus, evaluating the C5aR1–CXCL4 axis in this context may improve our understanding of disease mechanisms and provide new treatment options for COVID-19 and other diseases featuring thromboinflammation and complement involvement.

Complement-active drugs are available and already in use. 35 For instance, eculizumab, which inhibits the cleavage of C5 by the C5 convertase into C5a, has been clinically established for the treatment of aHUS (atypical hemolytic uremic syndrome) and for PNH (paroxysmal nocturnal hemoglobinuria). 36 Indeed, several novel pharmacological approaches have recently been introduced, i.e., Pegcetacoplan 37 or Avacopan, 38 a C5aR1 inhibitor, to the clinic. In light of our findings, implications of treatment regimen for vascular remodeling and regeneration should be assessed in future studies and should be taken into account when designing trials on complement therapeutics.

In conclusion, understanding the crosstalk of platelets with the complement system is important to apprehend the exact role of this interplay for platelet and complement activation and resulting diseases featuring thromboinﬂammation.
