# Supplementary material for: The Platelet Anaphylatoxin Receptor C5aR1 (CD88) Is a Promising Target for Modulating Vessel Growth in Response to Ischemia a
Source: TH Open. 2023 Oct 19;7(4):e289–93. doi: 10.1055/a-2156-8048 (PMC10586890; doi:10.1055/a-2156-8048)

Supplemental Figure 1

A

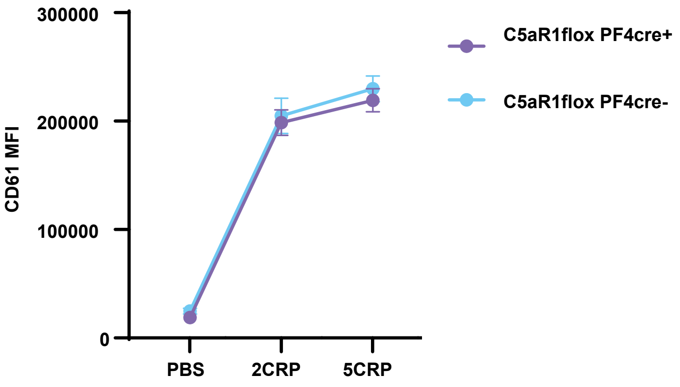

Supplemental Figure 2

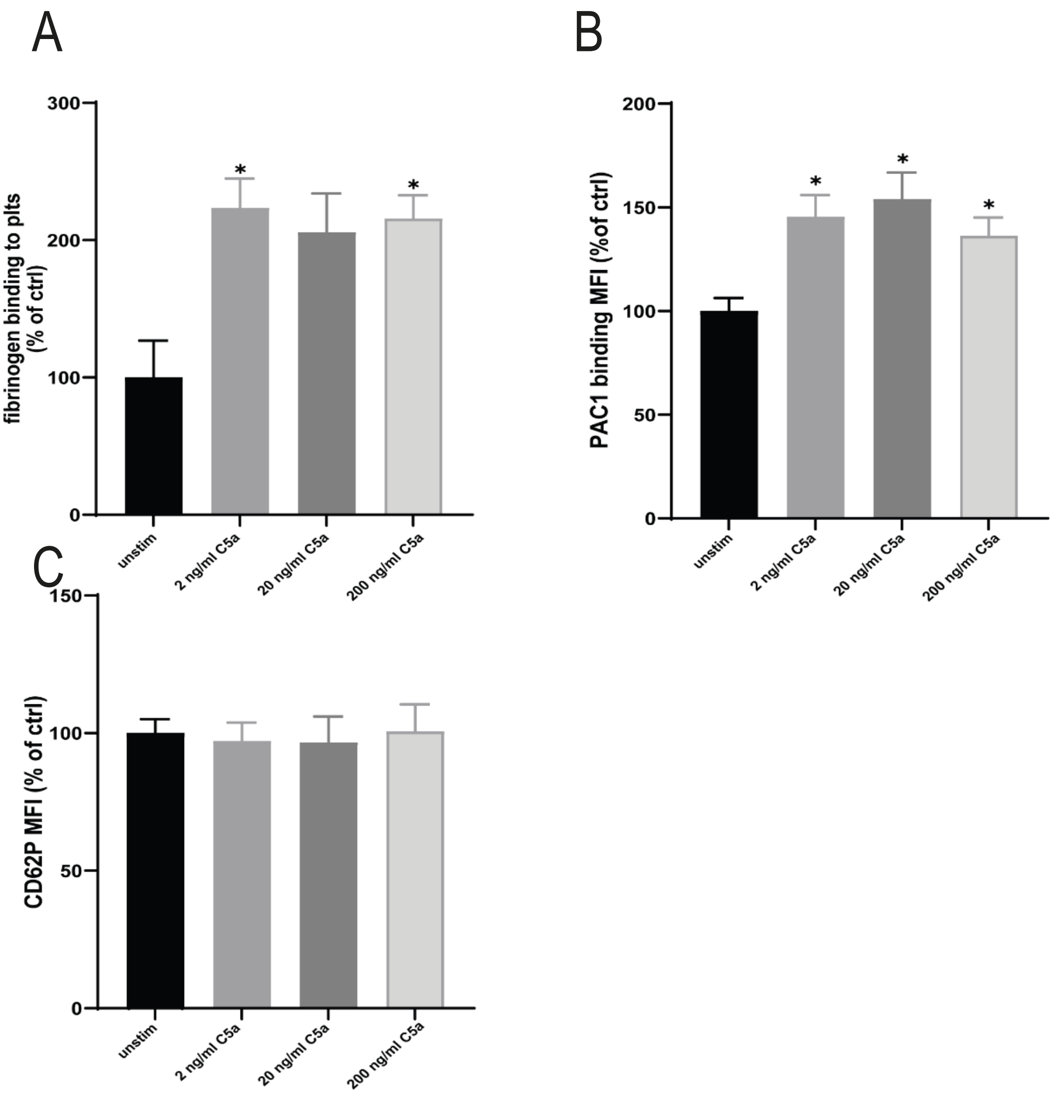

Supplemental Figure 3

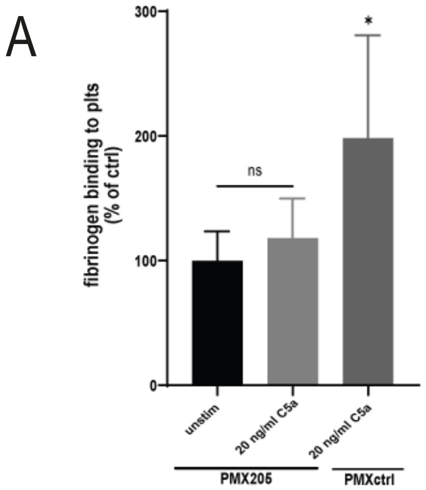

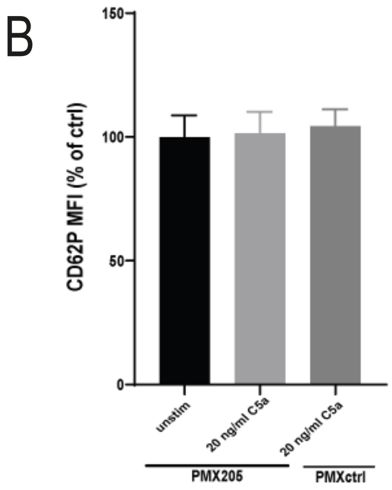

Supplemental Figure 4

A

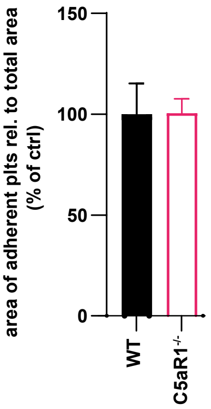

B

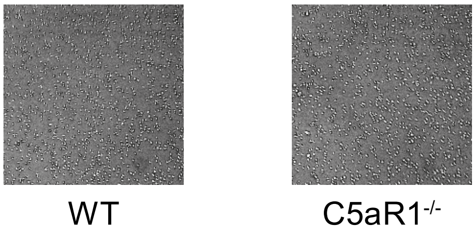

Supplemental Figure 5

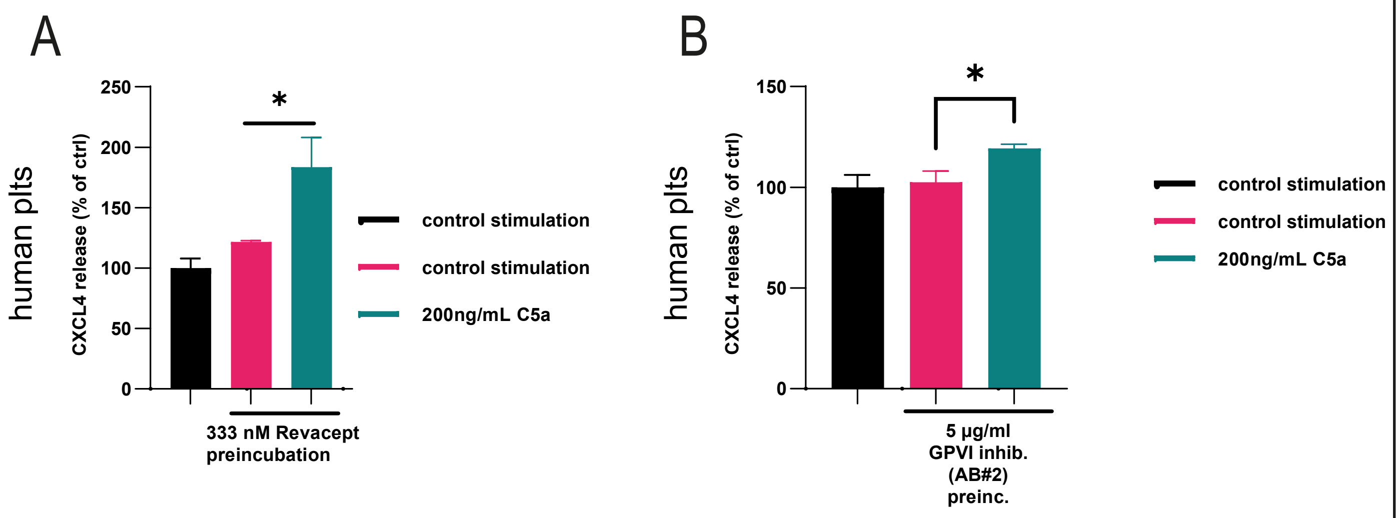

Supplement: Supplementary file 1 — Supplementary Material [file 10-1055-a-2156-8048-s23060023-1.pdf]
