# Supplementary material for: The Platelet Anaphylatoxin Receptor C5aR1 (CD88) Is a Promising Target for Modulating Vessel Growth in Response to Ischemia a
Source: TH Open. 2023 Oct 19;7(4):e289–93. doi: 10.1055/a-2156-8048 (PMC10586890; doi:10.1055/a-2156-8048)
Supplement: Supplementary file 2 — Supplementary Material [file 10-1055-a-2156-8048-s23060023-2.pdf]

Supplemental Information

Supplemental Methods

Mice

C57BL/6 (WT) mice were originally acquired from Janvier Labs but were bred in our own animal facility. Here, the light cycle was 12 h, temperature 20-22 °C, humidity 40-60%. C5aR1-deficient mice (CD88, C5aR1$^{-/-}$) have been previously described elsewhere (1); they were bred in our animal facility on the C57BL/6 background. GFP-C5aR1$^{fl/fl}$ mice have been described previously (2). Briefly, GFP-C5aR1–knock-in mice were generated by gene targeting. (Ac)GFP and an internal ribosomal entry site (IRES) were inserted adjacent to the coding exon of C5aR1. Simultaneously, the AcGFP IRES C5aR1 cassette was flanked with two loxP sites. We then crossed these mice with a PF4-cre strain expressing Cre-recombinase under control of the platelet- and megakaryocyte-specific promoter (3) to generate platelet-specific C5aR1-knockout mice.

Mouse hindlimb ischemia model

We used a previously described protocol to induce hindlimb ischemia (2,4). Briefly, the femoral artery of mice aged 10-12 weeks was ligated twice distal to the branch point of the caudal femoral artery and the epigastric artery. Afterwards, the femoral artery was intersected between both ligations. Tissue perfusion was assessed preoperatively, immediately post-ligation, and at 2, 4, 6, 8, 10 and 14 days after the surgical intervention, as previously described (5). Blood flow in the hindlimb was analyzed using an infrared laser Doppler imager (LDI2-IR, Moor Instruments, Axminster, UK) at 37°C under medetomidine/midazolam (0.5/5 mg/kg) anesthesia. Mice were left for a standard amount of time on the measuring heat mat to ensure consistency of results. Data were analyzed with Moor LDI image processing software (Moor Instruments) and reported as the ratio of flow in the ischemic versus nonischemic hindlimb. For the display of the LDI images, the colour scale was adapted to ensure maximum informativity of displayed results.

Flow cytometry

Platelets from mice were isolated as described below and stained with anti-mouse CD88-APC (C5aR1, clone 20/70, Biolegend, #135808) or rat IgG2bκ-APC Isotype Ctrl (clone RTK4530, Biolegend, #400612). Samples were analyzed with a FACSCalibur flow cytometer (Becton Dickinson, Heidelberg, Germany) and expressed as the mean fluorescence intensity (MFI) and data were analyzed using FloJo software (v.10, Tree Star, Ashland, OR, USA).

Most other flow cytometry experiments were performed with citrated whole blood, which was drawn from mice as described below. Blood was diluted 1:5 using Tyrode's solution (pH 7.4, supplemented with 1 mM $CaCl_2$ and 1 mM $MgCl_2$). In some experiments, blood was stimulated with the following agonists collagen related peptide (CRP; 2, 5 µg/ml; CambCol Laboratories, Cambridge, UK); C5a (20, 200 ng/ml; R&D). for 10 min at 37°C. After stimulation, blood was diluted 1:5 once again to stop activation and staining was performed at room temperature for 30 minutes using the following antibodies: PE/Dazzle™ 594 anti-mouse/rat CD61 Antibody, clone 2C9.G2, BioLegend, #104321). After staining, cells were fixed using freshly prepared 4% PFA solution and diluted using FACS buffer (PBS, 0.5% BSA, 0.1% Na-Azide).

For human platelets, staining was also performed in whole blood anticoagulated with CPDA (Citrate Phosphate Dextrose Adenine). For stimulation experiments of blood of healthy donors, we used recombinant human Complement Component C5a Protein (2, 20, 200 ng/ml); R&D) for 10 min at room temperature. For some experiment, platelets were preincubated with the C5aR1 antagonist PMX205 (used at 15 µM for 30 minutes at 37°C, Tocris Bioscience, R&D) or control peptide. Human PMX205 (Tocris Bioscience, R&D). The reaction was stopped by adding excess PBS. Staining was performed for 30 min at room temperature using the following antibodies: CD41 (CD41 PerCP/Cy5.5, clone HIP8, Biolegend, #303720) or CD41 APC, clone HIP8, Biolegend, #303710), anti-human CD62P Brilliant Violet 650 (P-Selectin, Biolegend), PAC-1 (CD41/CD61-FITC, clone PAC-1, Biolegend, #362804), fluorescently labelled fibrinogen (Fibrinogen from human Plasma, Alexa Fluor 546 conjugated, Thermo Fisher, #F13192), C5aR1 (CD88, clone P12/1). After staining, cells were fixed using PFA 0.5% and acquisition was performed on a Beckman Coulter Cytoflex S 4-laser cytometer (Beckman Coulter, Krefeld, Germany). Specific monoclonal antibody binding was expressed as the mean fluorescence intensity (MFI) of 50,000 platelets and data were analyzed using CytExpert software (v.2.4, Beckman Coulter).

The above-mentioned flow cytometry experiments were performed using a Beckman Coulter Cytoflex S 4-laser cytometer (Beckman Coulter, Krefeld, Germany) immediately after sample preparation and staining. Unless otherwise stated, specific monoclonal antibody binding was expressed as the geometric mean fluorescence intensity (geo. MFI) of 25,000 events in the target gate, and data were analyzed using CytExpert software (v.2.4, Beckman Coulter).

Human platelets from peripheral artery disease (PAD) patients were stained in whole blood within 20 minutes of blood withdrawal using CPDA tubes. Blood was drawn from patients suffering for asymptomatic PAD. All subjects consented freely to donate blood and all experiments were conducted in accordance with the specific ethical codes of the University of Lübeck, Germany. Asymptomatic PAD is defined as peripheral artery disease indicated by pathological ankle-brachial index below 0.9 as well as atherosclerotic arterial stenosis assessed by sonography, exclusion of diabetes mellitus and no walking pain in the patient history or during a six-minute walking test, respectively. Asymptomatic patients were matched with respect to gender and age with symptomatic patients Fontained stage IIb, verified by performing 6-minute walking test, during which all patients developed walking pain. Here, diabetes mellitus was excluded as well. Data were analyzed using Kaluza Analysis Software (ver. 2.1, Beckman Coulter). We measured C5aR1 expression in 24 patients. As C5aR1 should be quantified in resting platelets, we remove 4 patients with preactivated platelets from the analysis.

Immunofluorescence microscopy studies

From ischemic murine hindlimb muscle tissue, 8-µm-thick sections of the gastrocnemius muscle were processed for immunofluorescence staining. Matrigel sections or sections of human coronary thrombi were processed accordingly. Snap-frozen tissue was rehydrated in PBS, fixed for 10 min in ice-cold acetone and blocked with 10% goat or donkey serum and 1% BSA for 30 min at room temperature. Subsequently, the sections were incubated with primary antibody in 1% blocking serum containing CaCl2 and 0.2% Tween 20 overnight at 4°C.

As primary antibodies, we used goat anti-mouse C5aR1 antibody (CD88, Santa Cruz, clone P14, sc-3124, 1:200) and rat anti-mouse GPIb antibody (CD42b, clone POP-B, Emfret Analytics, 1:100). For visualization or secondary amplification, we incubated samples with donkey anti-goat Alexa Fluor 488 antibody (Invitrogen, 1:200) and goat

anti-rat cross-adsorbed IgG Alexa Fluor 594 (Thermo Fisher, 1:500). Finally, the sections were probed with DAPI (Sigma Aldrich, Taufkirchen, Germany, 1:3000) to visualize the nuclei.

The stained sections were mounted on glass slides, and image acquisition of muscle sections was performed as described earlier. Higher magnification images were taken using a Zeiss LSM 800 confocal laser scanning microscope with Zeiss ZEN 2.3 (blue edition) software. Subsequent image analysis was performed with Image Pro Plus (Ver. 7.0).

In vivo Matrigel plug assay

The in vivo Matrigel plug assay was performed as previously described (9) with some modifications. Two aliquots of Matrigel (0.5 ml, Corning, Tewksbury, MA, USA) containing recombinant hirudin (22.4 U/ml, Merck, Darmstadt, Germany) supplemented with basic fibroblast growth factor (bFGF, 150 ng/ml, PeproTech, Rocky Hill, NJ, USA) were subcutaneously injected into the mid-abdominal region of mice, one aliquot on each side. Hirudin was used as an anticoagulant because the classical anticoagulant heparin, that is usually used in Matrigel plug assays, has been shown to inhibit complement activation (10). In some experiments, Matrigel was supplemented with freshly isolated murine platelets (10^8/ml Matrigel) from various knockout mice, in addition to bFGF, as indicated in the figure legends. After 7 days, mice were sacrificed, and the Matrigel plugs were fixed with 4% PFA, processed for histology (frozen sections) and stained with H&E using standard staining protocols and reagents. Bright-field images were obtained with a Nikon Optishot-2 microscope equipped with a 2x plan-apochromat (N.A. 0.08) objective lens and a digital sight DS-5M camera using the Nikon NIS elements BR software (v.3.2, Nikon Instruments, Tokyo, Japan) for image acquisition and analysis. For readout, the ratio of the vessel area to the Matrigel plug area was calculated and expressed as neovascularization area fraction.

Isolation of human and murine platelets and generation of platelet releasate

Human and mouse washed platelets were isolated from human or mouse blood as previously described (16,17). Briefly, human venous blood was donated by healthy volunteers. All subjects consented freely to donate blood and all experiments were conducted in accordance with the specific ethical codes of the University of Tübingen and Lübeck, Germany. Blood was drawn from the antecubital vein into acid-citrate-

dextrose (ACD) buffer and centrifuged at 430x g for 20 min. PRP was removed and added to HEPES-buffered Tyrode's solution (2.5 mM HEPES, 150 mM NaCl, 1 mM KCl, 2.5 mM $NaHCO_3$, 0.36 mM NaH_2PO_4, 5.5 mM glucose and 1 mg/ml BSA, pH 6.5) and subsequently centrifuged at 900x g for 10 min. The resulting platelet pellet was resuspended in HEPES-buffered Tyrode's solution (pH 7.4, supplemented with 1 mM $CaCl_2$ and 1 mM $MgCl_2$). Subsequently, platelets were used for further experiments.
To isolate murine platelets, blood was drawn from the retroorbital plexus, collected in ACD buffer and centrifuged at 1800 rpm for 5min (Hettich EBA-3S, Germany). Supernatant was transferred, filled up with HEPES-buffered Tyrode's solution (pH7.4) and centrifuged at 800 rpm for 5min. Platelet rich plasma (PRP) was removed, filled up with Tyrode's solution then centrifuged at 2800 rpm for 5 min. The platelet pellet was carefully resuspended in HEPES-buffered Tyrode's solution (pH 7.4). The platelet content was quantified using a Sysmex cytometer (Sysmex KX-21N, Görlitz, Germany) and was adjusted to the required concentration. Subsequently, the platelets were either stimulated, and the supernatant was collected or lysed; the platelets were used for single-cell staining; or the platelets were used at specific concentrations for coincubation with endothelial cells, resuspended in Matrigel for injection into mice or used in flow cytometric analyses. For some experiments, platelet isolation was performed using activation inhibitors prostacyclin (0.5 µM, Sigma Aldrich) and apyrase (0.02U/mL, Sigma Aldrich).
As agonists for stimulation of murine as well as human platelets, we used C5a (R&D). For some experiments, platelets were preincubated with the C5aR1 antagonist PMX205 (used at 15 µM for 30 minutes at 37°C, Tocris) or control peptide. Subsequently, platelets were stimulated with C5a.
If not otherwise stated, stimulation with C5a (R&D) was performed at a concentration of 20 nM for 10 min at 37°C as previously described for other cell types (9). Following C5a stimulation, the supernatant was collected by centrifugation at 14,000x g at 4°C and analyzed or used to stimulate endothelial cells.

Flow chamber assay

Heparinized whole blood was perfused through a transparent flow chamber over a coated surface (fibrinogen (50 µg/ml; #341576, Merck Milipore) with moderate (1.000 s^{-1}) shear rates for 10 min (18). 3 reference images at 3 different positions were taken

using a a VWR IT414 microscope and the area of adherent platelets was quantified using ImageJ.

ELISA measurements

Platelet releasates were analyzed by ELISA using a mouse PF4/CXCL4 Quantikine ELISA Kit (R&D) as well as a human PF4/CXCL4 Quantikine ELISA Kit (R&D).

Scratched wound assay and tube formation assay

For the in vitro tube formation assay, MHEC-5T ($6x10^4$ cells/well) were plated onto Matrigel-coated (Corning, NY) Angiogenesis µ-slides (ibidi, Planegg, Germany) in endothelial culture medium containing 2% FBS. Cells were coincubated with platelet supernatant, recombinant mouse CXCL4 (Biolegend, San Diego, CA) or anti-mouse CD183 antibody (eBioscience, San Diego, CA, 10µg/ml). After 6h tube formation was imaged by phase-contrast microscopy. Tube Formation was analyzed using Angiogenesis analyzer for ImageJ as described before and total branching length as well as number of nodes per area were assessed (19). An example analysis is shown in Supplementary Fig. 11.

Legends to Supplemental Figures

Suppl. Fig. 1: Platelets of C5aR1$^{flox/flox}$PF4cre^{+} mice do not display a different level of reactivity

Citrated blood was drawn from mice, diluted and stimulate with CRP. Upon stimulation, both C5aR1$^{flox/flox}$PF4cre^{+} and C5aR1$^{flox/flox}$PF4cre^{-} display the same level of activation measured by CD61 upregulation. Data are displayed as the mean±SEM (n=6) and are shown as MFI *p<0.05.

Suppl. Fig. 2: Effect of C5a on activation of human platelets

Platelets were isolated from citrated whole blood of healthy donors. Subsequently, platelets were stimulated using different concentrations of C5a. (A) C5a induced a significant increase of fibrinogen binding of platelets. (B) We also found that C5a stimulation induced significant upregulation of activated GPIIIa (PAC-1) thus confirming this result. (C) However, no significant upregulation of CD62P was induced by C5a. Data are shown as the mean±SEM (n=4) and are displayed as a % of control. The mean MFI or the percent binding of platelets in the vehicle-stimulated group represents 100%.

Suppl. Fig. 3: Effect of C5aR1 blockade on C5a-induced activation of human platelets

Platelets were isolated from citrated whole blood of healthy donors. Subsequently, platelets were preincubated with C5aR1 antagonist PMX205 or the control peptide PMXctrl and then stimulated using C5a. (A) After PMX205 preincubation, C5a did not induce a significant increase of fibrinogen binding. (B) Also after PMX205-preincubation, C5a did not induce a change in CD62P expression. Data are shown as the mean±SEM (n=3-4) and are displayed as a % of control. The mean MFI or the percent binding of platelets in the vehicle-stimulated group represents 100%.

Suppl. Fig. 4: No phenotype of C5aR1-deficient platelets in adehiosn to fibrinogen under shear conditions.

Platelets isolated from WT or C5aR1$^{-/-}$ mice were perfused over coverslides coated with fibrinogen using a parallel-plate flow chamber. Under flow conditions (shear rate

1000s^{-1}), the adhesion of platelets was unaltered between both genotypes. Data represents mean±SEM. n=4-5. *p<0.05.

Suppl. Fig. 5: Platelets C5aR1-mediated CXCL4 release is not dependent on GPVI

(A) Washed human platelets were stimulated with C5a. The supernatant was analyzed by ELISA for the level of CXCL4. Preincubation with Revacept (kindly provided by Götz Münch) did not inhibit C5a-induced CXCL4 secretion. Data are shown as the mean±SEM. n= 4. *p<0.05.

(B) Washed human platelets were stimulated with C5a. The supernatant was analyzed by ELISA for the level of CXCL4. Preincubation with an antiGPVI antibody (kindly provided by Götz Münch) decreased but did not inhibit C5a-induced CXCL4 secretion. Data are shown as the mean±SEM. n= 3-4. *p<0.05.

Supplemental References

1. Hopken UE, Lu B, Gerard NP, et al. The C5a chemoattractant receptor mediates mucosal defence to infection. Nature 1996; 383: 86–9.
2. Nording H, Baron L, Haberthür D, et al. The C5a/C5a receptor 1 axis controls tissue neovascularization through CXCL4 release from platelets. Nat Commun Nature Publishing Group; 2021; 12: 3352.
3. Tiedt R, Schomber T, Hao-Shen H, et al. Pf4-Cre transgenic mice allow the generation of lineage-restricted gene knockouts for studying megakaryocyte and platelet function in vivo. Blood 2007; 109: 1503–6.
4. Limbourg A, Korff T, Napp LC, et al. Evaluation of postnatal arteriogenesis and angiogenesis in a mouse model of hind-limb ischemia. Nat Protoc Nature Publishing Group; 2009; 4: 1737–48.
5. Qi X, Yuan Y, Xu K, et al. (2-Hydroxypropyl)-β-cyclodextrin is a new angiogenic molecule for therapeutic angiogenesis. PLoS One 2015; 10: 1–16.
6. Li Y, Sun J-F, Cui X, et al. The effect of heparin administration in animal models of sepsis: a prospective study in Escherichia coli-challenged mice and a systematic review and metaregression analysis of published studies. Crit Care Med NIH Public Access; 2011; 39: 1104.
7. Patzelt J, Mueller KAL, Breuning S, et al. Expression of anaphylatoxin receptors on platelets in patients with coronary heart disease. Atherosclerosis 2015; 238: 289–95.
8. Geue S, Aurbach K, Manke M-C, et al. Pivotal Role of PDK1 in Megakaryocyte Cytoskeletal Dynamics and Polarization during Platelet Biogenesis. Blood 2019; 134: 1847–58.
9. Langer HF, Chung KJ, Orlova V V., et al. Complement-mediated inhibition of neovascularization reveals a point of convergence between innate immunity and angiogenesis. Blood 2010; 116: 4395–403.
10. Girardi G, Redecha P, Salmon JE. Heparin prevents antiphospholipid antibody-induced fetal loss by inhibiting complement activation. Nat Med 2004; 10: 1222–6.
11. Langer HF, Choi EY, Zhou H, et al. Platelets Contribute to the Pathogenesis of Experimental Autoimmune Encephalomyelitis. Circ Res 2012; 110: 1202–10.
12. Schleicher RI, Reichenbach F, Kraft P, et al. Platelets induce apoptosis via

membrane-bound FasL. Blood 2015; 126: 1483–93.

13. Sauter RJ, Sauter M, Reis ES, et al. Functional Relevance of the Anaphylatoxin Receptor C3aR for Platelet Function and Arterial Thrombus Formation Marks an Intersection Point Between Innate Immunity and Thrombosis. Circulation 2018; 138: 1720–35.
14. Sauter R, Sauter M, Obrich M, et al. Anaphylatoxin Receptor C3aR Contributes to Platelet Function, Thrombus Formation and In Vivo Haemostasis. Thromb Haemost 2019; 119: 179–82.
15. Abe T, Hosur KB, Hajishengallis E, et al. Local Complement-Targeted Intervention in Periodontitis: Proof-of-Concept Using a C5a Receptor (CD88) Antagonist. J Immunol Am Assoc Immnol; 2012; 189: 5442–8.
16. Nording H, Giesser A, Patzelt J, et al. Platelet bound oxLDL shows an inverse correlation with plasma anaphylatoxin C5a in patients with coronary artery disease. Platelets 2016; 27: 593–7.
17. Langer H, May AE, Daub K, et al. Adherent platelets recruit and induce differentiation of murine embryonic endothelial progenitor cells to mature endothelial cells in vitro. Circ Res 2006; 98: e2–10.
18. Langer HF, Daub K, Braun G, et al. Platelets Recruit Human Dendritic Cells Via Mac-1/JAM-C Interaction and Modulate Dendritic Cell Function In Vitro. Arterioscler Thromb Vasc Biol 2007; 27: 1463–70.
19. Carpentier G, Martinelli M, Courty J, et al. Angiogenesis analyzer for ImageJ. In4th ImageJ User Dev Conf Proc 2012. p. 198–201.
